# Molecular Design Strategies of Nucleating Agents with Synergistic Effects for Upcycling Polyethylene Terephthalate

**DOI:** 10.3390/molecules31030414

**Published:** 2026-01-26

**Authors:** Xinyu Hao, Tianjiao Zhao, Fuhua Lin, Meizhen Wang, Dingyi Ning, Wenju Cui, Yuanjian Ye, Jun Luo, Bo Wang

**Affiliations:** 1 School of Chemical Engineering and Technology, Taiyuan University of Science and Technology, Taiyuan 030024, China; s202421211097@stu.tyust.edu.cn (X.H.); s202221211044@stu.tyust.edu.cn (T.Z.); s202321111073@stu.tyust.edu.cn (M.W.); ningdingyi@tyust.edu.cn (D.N.); cuiwenju@tyust.edu.cn (W.C.); 2 School of Traffic Engineering, Shanxi Vocational University of Engineering Science and Technology, Jinzhong 030619, China; linfuhua@sxgkd.edu.cn; 3 Guangzhou Quality Testing & Inspection Institute, Guangzhou 511400, China; yeyuanjian@gmail.com (Y.Y.); luoj@iccas.ac.cn (J.L.)

**Keywords:** recycled polyethylene terephthalate, lubrication effect, compatibility, crystallization behavior, mechanical properties

## Abstract

The nucleating agents with different alkyl chain lengths sodium 4-[(benzyl)amino] benzoate (SAB-Be), sodium 4-[(heptanoyl)amino] benzoate (SAB-7C), and sodium 4-[(stearoyl)amino] benzoate (SAB-18C) were synthesized via chemical to improve the crystallization and mechanical properties of recycled polyethylene terephthalate (rPET) that had been damaged during mechanical recycling. The rPET/nucleating agent blends were prepared by melt blending. The molecular structure and thermal stability of the nucleating agents were characterized using the utilization of fourier transform infrared (FTIR) and thermogravimetric analysis (TGA). The differential scanning calorimetry (DSC) results showed that the crystallization properties of the rPET had been improved. In addition, the glass transition temperatures (*T*_g_) of rPET, rPET/SAB-Be, rPET/SAB-7C, and rPET/SAB-18C were 80.3 ± 0.3 °C, 80.4 ± 0.9 °C, 77.0 ± 1.2 °C, and 69.7 ± 0.9 °C, respectively, demonstrating that the length of the alkyl chain in the nucleating agents was essentially proportional to the lubrication effect on rPET. Meanwhile, the rheological properties also supported the conclusion. The isothermal thermodynamic analysis indicated that the compatibility between nucleating agents and rPET was related to the length of the alkyl chain in the nucleating agents. The scanning electron microscopy (SEM) results of the fracture surfaces of the rPET/nucleating agent blends showed that the longer the alkyl chain in the nucleating agent, the greater the compatibility with rPET. Furthermore, the rPET/SAB-18C exhibited the best mechanical properties of the samples used in this research, with flexural strength and impact strength increased by 5.1% and 58.9%, respectively, compared to rPET. Overall, this work provided the new approach for rPET upcycling by combining molecular design strategies.

## 1. Introduction

Polyethylene terephthalate (PET) is a type of polyester with excellent physical and chemical properties, including high resistance to organic solvents, durability, and thermal stability. Consequently, PET has been widely used in packaging materials and textile applications [1,2,3,4]. With annual production exceeding 70 million tons, the non-degradable PET has caused serious environmental pollution [5]. Thus, effective PET recycling methods are being intensively developed [6]. Currently, recycled polyethylene terephthalate (rPET) is mainly obtained through mechanical and chemical recycling [2]. The main steps of mechanical recycling include shredding, melting, and reforming, which are characterized by simple operation and economic efficiency. Therefore, mechanical recycling is the most widely used method for PET recycling [7,8].

However, the thermal processing involved in mechanical recycling leads to the degradation of macromolecules, which adversely affects the crystallization and mechanical properties of rPET and limits the range of applications for rPET [2,9,10,11]. To overcome the problem, blending modification is considered a practical and effective method for improving the crystallization behavior and mechanical properties of rPET [12]. Blending with nucleating agents can reduce the nucleation energy barrier for rPET crystallization through the heterogeneous nucleation, which improves the degree of molecular chain ordering compromised by macromolecular degradation, thereby increasing the crystallization temperature and shortening the molding cycle [13]. Additionally, the incorporation of nucleating agents can optimize the crystal structure of rPET, thereby enhancing the mechanical properties of rPET [14].

As nucleating agents provide external surfaces for polymers, the compatibility between nucleating agents and the polymer matrix is critical [15]. Currently, nucleating agents used to modify rPET are mainly organic small molecules and inorganic particles [16]. Common inorganic particles include clays [17,18], calcium carbonate [19], and silica [20]. Due to the tendency of inorganic particles with high specific surface energy to aggregate in the rPET matrix during the melt blending process [21], the organic small molecules with high nucleating efficiency and good compatibility with rPET have become the most promising nucleating agents for blending modification of rPET. At present, the commonly used organic small molecule nucleating agents for modified PET mainly include sodium benzoate (SB) [22], dibenzylidene sorbitol (DBS) [23], and sodium linoleate (Na-LL) [24]. However, the melting temperatures of DBS (224 °C) and Na-LL (192 °C) are both lower than the processing temperature of rPET (270 °C), which can cause chemical nucleation during rPET processing and result in a decrease in its molecular weight. As a result, the modification effect of nucleation agents could be limited. SB has the potential to cause degradation of PET and subsequently affect the mechanical properties [25]. Therefore, it is necessary to find an organic small molecule with high thermal stability and good compatibility with the rPET matrix to serve as the dispersing nucleating agent.

The effectiveness of organic nucleating agents is related to the chemical structure. Nam et al., [26] modified polylactic acid (PLA) with the N,N-ethylenebis (12-hydroxystearamide) (WX1) and the results demonstrated that the crystallization rate of PLA was significantly increased by the addition of WX1. Furthermore, the research concluded that amide compounds containing specific polar functional groups (-NH) can serve as effective nucleating agents for polyesters. In light of the finding, Shen et al., [27] employed a polypropylene (PP) specific nucleating agent N,N’-dicyclohexyltereph-thalamide (TMB-5) to improve the crystallization and mechanical properties of PET. With the addition of TMB-5 at 0.6 wt%, the crystallization temperature of PET increased by 16.6 °C and the impact strength increased by ten-fold. In our previous work, the impact of the sodium 4-[(4-chlorobenzoyl)amino]glycine (Na-4-ClBeAmGl) which comprises aromatic amide structures on rPET was investigated [28]. The results showed that the addition of Na-4-ClBeAmGl at 0.5 wt% resulted in 22.6 °C increase in the crystallization temperature of rPET and 13.6% enhancement in impact strength.

However, Dong et al., [16] found that the organic small molecule nucleating agent sodium 4-[(4-chlorobenzyl)amino]benzoate (SCAB) exhibited agglomeration in the PP matrix. To address this issue, the zinc stearate (Znst), which contains long alkyl chains, was used as lubricant to improve the compatibility between SCAB and PP. The results showed that Znst was unable to facilitate the crystallization of PP, but the incorporation provided internal lubrication role and enabled the uniform dispersion of SCAB within the PP matrix. Compared with PP/SCAB, the crystallization and mechanical properties of the PP/SCAB-Znst were significantly improved. Tang et al. [29] employed a low molecular weight styrene body acrylic ionomer (SAA-Na), containing long alkyl chains to blend with PET. The results indicated that SAA-Na exhibited favorable compatibility with PET and the crystal structure of the PET/SAA-Na was more perfect than PET.

The above research indicates that additives with long alkyl chains have good compatibility with polymers. However, current nucleating agents for rPET rarely combine long alkyl chains with aromatic amide structures, and the synergistic effect of such structural features on lubrication, compatibility, crystallization kinetics, and mechanical properties has been scarcely reported. Therefore, this study adopts a molecular design approach, using aromatic amide nucleating agents as the base material. By controlling the alkyl chain length, it elucidates how the improved compatibility between aromatic amide nucleating agents with varying alkyl chain lengths and rPET influences their crystallization behavior and mechanical properties.

In this study, three aromatic amide nucleating agents with different alkyl chain lengths were synthesized by chemical reactions. The molecular structure and thermal stability of the nucleating agents were investigated by fourier transform infrared (FTIR) and thermogravimetric analysis (TGA). Three types of nucleating agents were melt-blended with rPET to prepare the rPET/nucleating agent blends. The crystallization behavior and mechanism of the rPET/nucleating agent blends were investigated by differential scanning calorimeter (DSC). The rheological behavior of the rPET/nucleating agent blends was analyzed to describe the lubrication effect of the nucleating agents. The mechanical properties of the rPET/nucleating agent blends were also studied. Finally, the compatibility of the nucleating agents in the rPET matrix was observed using scanning electron microscope (SEM).

## 2. Results and Discussion

### 2.1. FTIR and TGA of the Nucleating Agents

**Figure 1:**
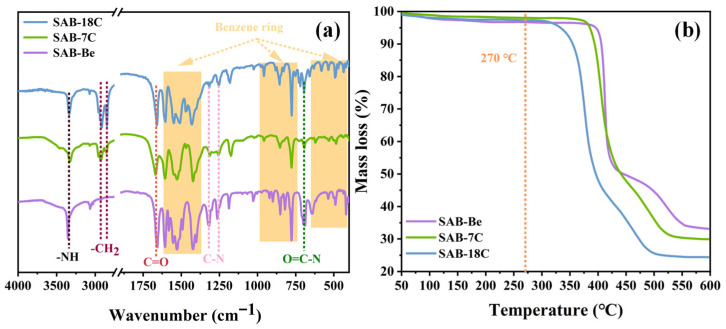
(**a**) FTIR spectra of the nucleating agents; (**b**) TG curve of the nucleating agents.

The FTIR spectra of the nucleating agents are shown in Figure 1a. The vibration peaks at 3361 cm^−1^, 1660 cm^−1^, and 680 cm^−1^ represented the -NH, C=O, and O=C-N groups in aromatic amides, which proved the amidation reaction between PABA and acyl chloride has occurred successfully. The characteristic peaks at 1606 cm^−1^, 1529 cm^−1^, and 1423 cm^−1^ were attributed to the vibrations of the benzene ring skeleton. The characteristic peaks of C-H on the benzene ring appear at 900–680 cm^−1^. Meanwhile, the vibrations peaks at 660–400 cm^−1^ represent the in-plane and out-of-plane ring deformation vibration of the benzene ring. In addition, the vibration peaks at 1325 cm^−1^ and 1261 cm^−1^ represented the stretching vibration of C-N. The characteristic peaks at 2922 cm^−1^ and 2853 cm^−1^ represent the asymmetric and symmetric stretching vibrations of -CH_2_. As shown in Figure 1a, only SAB-7C and SAB-18C exhibit the characteristic peak of -CH_2_, with SAB-18C displaying more pronounced peaks than SAB-7C, indicating that SAB-18C has a longer alkyl chain. The analysis of the FTIR spectra confirms that the nucleating agents were synthesized successfully.

Figure 1b shows the TG curve of the nucleating agents. The samples did not exhibit significant mass loss up to 300 °C. This result suggests that the nucleating agents with different alkyl chain lengths all have great thermal stability and do not decompose within the processing temperature range of rPET (270 °C). Therefore, all the samples can be used as dispersing nucleating agents for rPET. In addition, the DSC results of the nucleating agent showed that only SAB-18C exhibited a melting peak at 275 °C, while SAB-Be and SAB-7C did not melt at 300 °C.

### 2.2. Non-Isothermal Crystallization Behavior Analysis of the rPET/Nucleating Agent Blends

**Figure 2:**
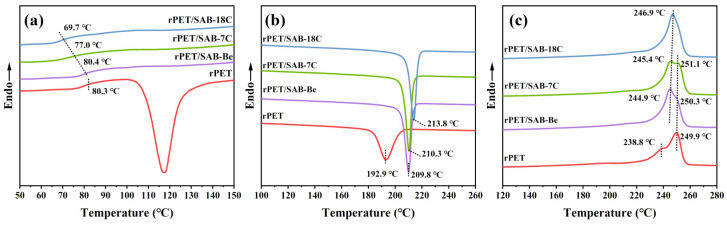
(**a**) First heating thermogram of the blends; (**b**) crystallization thermogram of the blends; and (**c**) second heating thermogram of the blends.

The first heating thermogram of the rPET/nucleating agent blends are shown in Figure 2a. It can be seen that rPET exhibits the clear cold crystallization peak (*T*_cc_) while the addition of the nucleating agents leads to the disappearance of *T*_cc_. The formation of *T*_cc_ is attributed to the inability of rPET molecular chains to form an ordered structure in time during rapid cooling to room temperature after melt processing [30]. Therefore, the phenomenon illustrates that the addition of nucleating agents can promote the movement of the rPET molecular chains and make the rPET molecular chains easier to stack, thereby improving the crystallization ability of rPET during the rapid cooling process [31].

The glass transition temperature (*T*_g_) of the rPET/nucleating agent blends is shown in Table 1. Compared with rPET, the *T*_g_ of rPET/SAB-Be showed almost no change, while the *T*_g_ of rPET/SAB-7C and rPET/SAB-18C decreased by 3.3 °C and 10.6 °C, respectively. The result indicates that the long alkyl chains in SAB-7C and SAB-18C provide a lubricating effect, thereby enhancing the mobility of rPET molecular chains [16]. Compared to SAB-7C, the SAB-18C further increases the mobility of rPET molecular chains and lowers their glass transition temperature due to the longer alkyl chains. This may be attributed to SAB-18C achieving a certain effect of plasticizing in rPET [32].

The crystallization curve of the rPET/nucleating agent blends is shown in Figure 2b, and the data are recorded in Table 1. It can be observed that compared with rPET, the crystallization temperature (*T*_cp_) of rPET/SAB-Be, rPET/SAB-7C, and rPET/SAB-18C increased by 16.9 °C, 17.4 °C, and 20.9 °C, respectively. The result indicates that the nucleating agents can promote the onset of crystal growth and shorten the forming cycle of rPET [33]. In addition, SAB-18C with the longest alkyl chains can significantly improve the crystallization ability of the rPET. The reason for this phenomenon may be that SAB-18C has the best compatibility with rPET.

Figure 2c shows the second heating thermogram of the rPET/nucleating agent blends. The melting peak of rPET consisted of a low-temperature shoulder peak and one high-temperature main peak [34]. The phenomenon was due to the following two processes during the second melting of rPET: melting of crystals formed during the crystallization and melting of crystals formed during recrystallization [35]. The melting of the crystals formed during primary crystallization generated the low-temperature shoulder peak (*T*_m1_), while the high-temperature peak (*T*_m2_) corresponded to the melting of the crystals formed as a result of recrystallization during heating [29,36]. Compared with rPET, the addition of nucleating agents significantly increased *T*_m1_. According to the Hoffman–Lauritzen theory, the increase in melting temperature (*T*_m_) was attributed to the thickening of the polymer lamellae [37]. Therefore, the results indicate that as the alkyl chain length of the nucleating agents increased, the compatibility with rPET improved, leading to the stronger ability to induce the regular arrangement of the rPET molecular chains and the formation of a more perfect crystal structure in rPET. The *T*_m2_ of rPET/SAB-Be and rPET/SAB-7C was slightly higher than that of rPET, which was also attributed to the increased lamella thickness in rPET/SAB-Be and rPET/SAB-7C. However, it can be clearly observed from Figure 2c that the rPET/nucleating agent blends substantially reduced the proportion of the recrystallization process compared with rPET, and rPET/SAB-18C almost did not undergo the melting process during the recrystallization stage. The result indicates that the addition of nucleating agents reduced the imperfect crystal structure of rPET. Meanwhile, SAB-18C had the strongest ability to improve the crystal structure of rPET during the crystallization process. Compared with rPET, the crystallinity (*X*_c_) of rPET/SAB-Be, rPET/SAB-7C, and rPET/SAB-18C increased by 5.5%, 5.8%, and 7.1%, respectively, which clearly reflects the ability of SAB-Be, SAB-7C, and SAB-18C to improve the crystal structure of rPET.

### 2.3. Isothermal Crystallization Kinetic Analysis of the rPET/Nucleating Agent Blends

**Figure 3:**
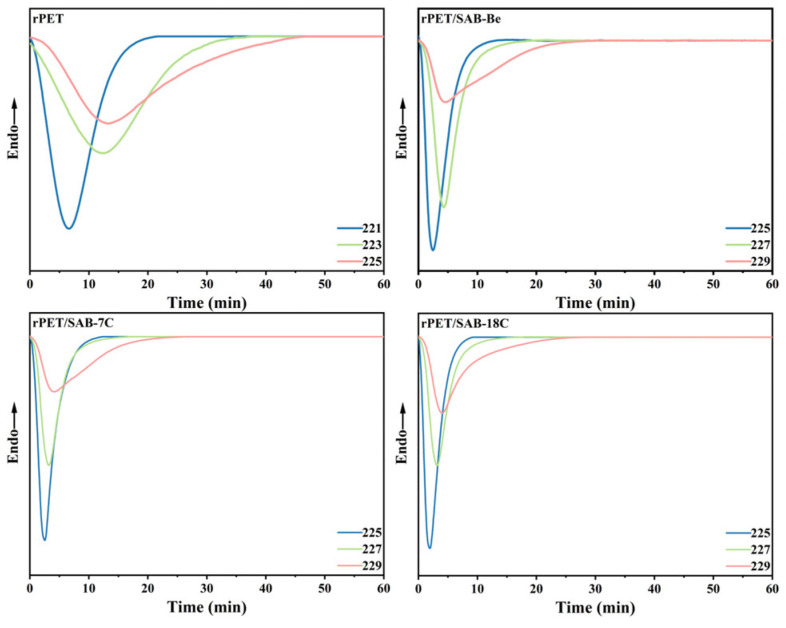
Isothermal crystallization thermogram of the rPET/nucleating agent blends.

To ensure clearer observation of the crystallization process during experiments and to guarantee data accuracy, the isothermal crystallization behavior of rPET was investigated by selecting isothermal crystallization temperatures (*T*_c_) of 221 °C, 223 °C, and 225 °C. As the *T*_cp_ of the rPET/nucleating agent blends was higher than that of rPET, the *T*_c_ of 225 °C, 227 °C, and 229 °C were selected. The curves are shown in Figure 3. It can be seen that the peak width is related to the crystallization rate. This rate is higher at low temperatures (with a sharp peak) and lower at higher temperatures (with a wider peak). This is because increasing the crystallization temperature inhibits the adsorption and folding growth of the rPET molecular chains on the surface of the crystal nucleus, leading to a reduction in the nucleation and growth rate of rPET [21].

The Avrami equation is an empirical equation that establishes a relationship between the degree of polymer crystallization to the crystallization time [38]. The general form is as follows:

(1) $$X_{t}\;=\;\exp\left(-K_{t}t^{n}\right)$$

where *X_t_* represented the relative crystallinity at time *t*, *K* represented the crystallization rate constant which included both nucleation and growth rates, and *n* represented the Avrami index which was related to the nucleation and crystal growth modes throughout the rPET crystallization process.

At the same time, *X_t_* can be represented as follows:

(2) $$X_{t}\;=\;\frac{\int_{t_{0}}^{t}\frac{dH_{c}}{dt}dt}{\int_{t_{0}}^{t_{e}}\frac{dH_{c}}{dt}dt}$$

**Figure 4:**
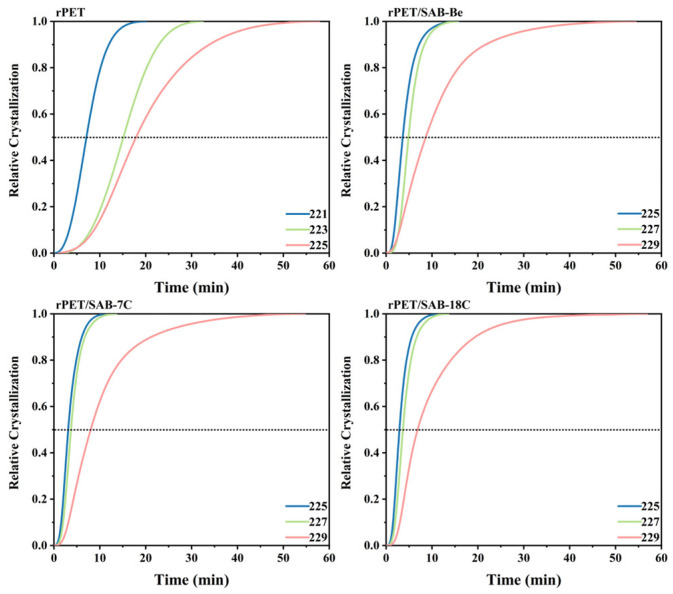
Relative degree of crystallinity with time of the rPET/nucleating agent blends.

Equation (2) was used to convert the isothermal crystallization thermogram (Figure 3) into the relationship between *X_t_* and *t* (Figure 4), the crystallization time corresponding to *X_t_* = 0.5 was *t*^a^_1/2_ (Table 2), which was the actual time when crystallization was 50% complete.

The *t*^a^_1/2_ of rPET, rPET/SAB-Be, rPET/SAB-7C, and rPET/SAB-18C at 225 °C was 17.6 min, 3.6 min, 3.1 min, and 2.9 min, respectively. The results indicate that the addition of the nucleating agents can effectively reduce the time required for crystallization and accelerate the crystallization rate of rPET. In addition, *t*^a^_1/2_ increased with increasing *T*_c_ which was consistent with the trend of peak width in the isothermal crystallization thermogram. The *t*^a^_1/2_ of rPET/SAB-Be, rPET/SAB-7C, and rPET/SAB-18C at 229 °C was 8.6 min, 7.4 min, and 6.0 min, respectively. The result demonstrates that the compatibility between nucleating agents and rPET improved as the alkyl chain length of the nucleating agents increased, thereby promoting the stacking and growth of the rPET molecular chains on heterogeneous nucleation surfaces.

In order to facilitate further plotting, the logarithm of both sides of Equation (1) was taken to obtain Equation (3).

(3) $$\ln[-\ln(1-X_{t})]=n\ln t+\ln K$$

**Figure 5:**
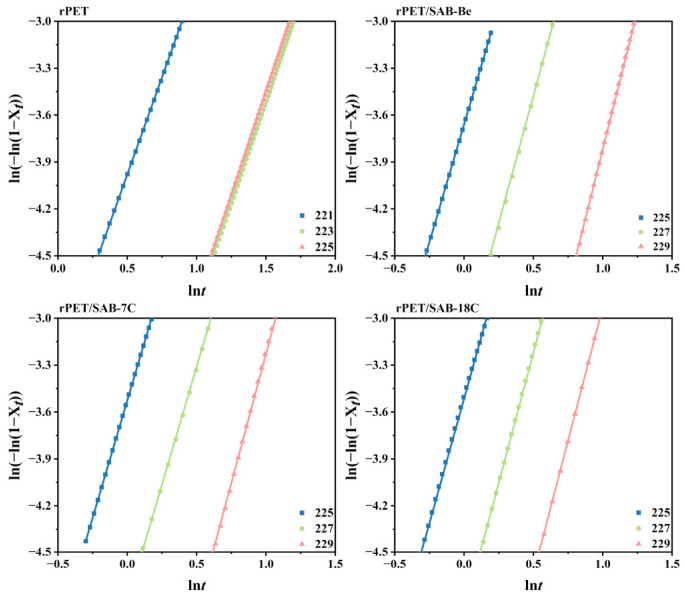
Isothermal crystallization kinetics curves of the rPET/nucleating agent blends.

According to Equation (3), ln[−ln(1 − *X_t_*)] was plotted against ln*t* and fitted to obtain a straight line (Figure 5) with a slope of *n* and an intercept of *K* (Table 2). Figure 5 shows that the ln[−ln(1−*X_t_*)] for the samples had good linear relationship with ln*t*, indicating that the Avrami equation was applicable to the samples. The *K* decreased with increasing *T*_c_ for the same sample. Meanwhile, the rPET/SAB-18C had the highest *K* value at the same *T*_c_, also indicating that SAB-18C had the strongest ability to accelerate the crystallization rate of rPET. Table 2 shows that the *n* value for all samples was close to three. The result indicates that the crystal growth mode changed from two-dimensional to three-dimensional growth with the addition of the nucleating agents.

The *t*^b^_1/2_ which corresponded to the completion of 50% of the theoretical crystallization time was calculated according to Equation (4):

(4) $$t_{1/2}^{b}\;=\;{\left(\frac{\ln2}{K}\right)}^{1/n}$$

The values of *t*^a^_1/2_ and *t*^b^_1/2_ were essentially identical, as shown by the data presented in Table 2, thereby confirming the applicability of the Avrami equation to the samples.

### 2.4. Isothermal Crystallization Thermodynamic Analysis of the rPET/Nucleating Agent Blends

**Figure 6:**
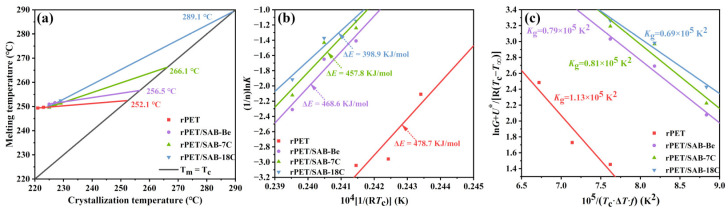
(**a**) *T*_m_ versus *T*_c_ for the rPET/nucleating agent blends; (**b**) (1/*n*)ln*K* versus 1/(R*T*_c_) for the rPET/nucleating agent blends; and (**c**) ln*G* + $\frac{U^{*}}{R(T_{c}-T_{\infty })}$ versus $\frac{1}{T_{c}∆Tf}$ for the rPET/nucleating agent blends.

*T*_m_ was plotted against *T*_c_ to obtain a straight line, which was then extrapolated towards *T*_m_ = *T*_c_ to determine the apparent *T*^0^_m_ of the sample (Figure 6a). The data are presented in Table 3. The *T*^0^_m_ denotes the melting temperature at which the polymer molecular chain reaches thermodynamic equilibrium when fully extended [39,40]. Compared with rPET, the apparent *T*^0^_m_ of rPET/SAB-Be, rPET/SAB-7C, and rPET/SAB-18C increased by 4.4 °C, 14.0 °C, and 37.0 °C, respectively. According to the Gibbs–Thomson theory [41], the thickness of polymer lamellae is proportional to *T*^0^_m_, thereby indicating that the order of crystal perfection for each sample is as follows: rPET/SAB-18C > rPET/SAB-7C > rPET/SAB-Be. This is because nucleating agents with longer alkyl chains exhibit better compatibility with rPET, which can effectively improve the crystal structure of rPET, and increase the lamellae thickness.

The Arrhenius equation can be used to calculate the crystallization activation energy (Δ*E*) of the polymers [42]:

(5) $$K^{1/n}\;=\;k_{0}\exp\left(-\frac{∆E}{RT_{c}}\right)$$

where *k*_0_ was the temperature dependent pre-exponential coefficient, *R* was the gas constant and typically taken as 8.314 J/(mol·K), and Δ*E* was the crystallization activation energy.

According to Equation (5), (1/*n*)ln*K* was plotted against 1/(*RT*_c_) and linear fitting was performed to obtain the slope Δ*E*. As shown in Figure 6b and Table 3, compared with rPET, the Δ*E* of rPET/SAB-Be, rPET/SAB-7C, and rPET/SAB-18C decreased by 10.1 KJ/mol, 20.9 KJ/mol, and 79.8 KJ/mol, respectively. Δ*E* represents the energy barriers that polymers must overcome to transition from the amorphous to the crystalline state. The value of Δ*E* reflects the difficulty of the polymer crystallization that the reduction in Δ*E* facilitates the crystallization of polymers. Therefore, the results suggest that the addition of the nucleating agents can significantly reduce the energy required for rPET crystal growth. The lowest ΔE value for rPET/SAB-18C indicates that the long alkyl chain of SAB-18C exerts an internal lubricating effect within rPET. This weakens the intermolecular forces in rPET, accelerates its relaxation behavior, and thereby reduces the potential energy barrier during crystal growth.

The Avrami equation describes the overall crystal behavior of polymers, including nucleation and growth. The Lauritzen–Hoffman equation provides a description of the nucleation process during spherulite growth [43]:

(6) $$G\;=\;G_{0}\exp(-\frac{U^{*}}{R(T_{c}-T_{\infty })})\exp(-\frac{K_{g}}{T_{c}\;\times \;\Delta T\;\times \;f})$$

where *G*_0_ referred to the pre-factor, while U* represented the migration activation energy, which was 6.28 KJ/mol. *f* = 2*T*_c_/((*T*^0^_m_ + *T*_c_)), was the melting heat correction temperature. The degree of undercooling was given by Δ*T* = *T*^0^_m_ − *T*_c_. *T*_∞_ = *T*_g_ − *C*, was expressed as the characteristic temperature of the polymer, where *C* = 30 K, *K*_g_ was the nucleation parameter which varies with different nucleation methods, and Equation (7) was as follows [44]:

(7) $$K_{g}\;=\;\frac{4b_{0}\sigma \sigma_{e}T_{m}^{0}}{∆\mathrm{Hk}}$$

where *σ* = 0.1*b*_0_∆*H* represented the surface free energy per unit area of the folded chain side. For rPET, *b*_0_ was 0.595 nm, representing the thickness of the monolayer. The enthalpy of the melting per unit volume was ∆*H* = 2.1 × 108 J/m^3^, *K* = 1.38 × 10^−23^ J/K, which was the Boltzmann constant. *σ*_e_ was the surface free energy per unit area of the molecular chain end.

According to Equation (6), 1/(*T*_c_Δ*Tf*) was plotted against ln*G* + U*/(*R*(*T*_c_ − *T*_∞_)). The slope of the linear fit was *K*_g_ and the value of *σ*_e_ was subsequently calculated using *K*_g_. The data are presented in Figure 6c and Table 3. Compared with rPET, the *K*_g_ and *σ*_e_ of the rPET/nucleating agent blend decreased significantly, indicating that the addition of nucleating agent can effectively reduce the energy required for rPET nucleation and growth. Among these, the *K*_g_ and *σ*_e_ of rPET/SAB Be and rPET/SAB-7C were not significantly different, while the *K*_g_ and *σ*_e_ of rPET/SAB-18C were the lowest, reduced by 0.44 × 10^5^ K^2^ and 8.2 erg/cm^2,^ respectively, compared to rPET. The result indicated that the significant increase in the alkyl chain length of the nucleating agent can effectively reduce the energy barrier that rPET must overcome during the nucleation process and promote the crystallization growth of rPET from the thermodynamic perspective.

### 2.5. Polarized Optical Microscopy of the rPET/Nucleating Agent Blends

**Figure 7:**
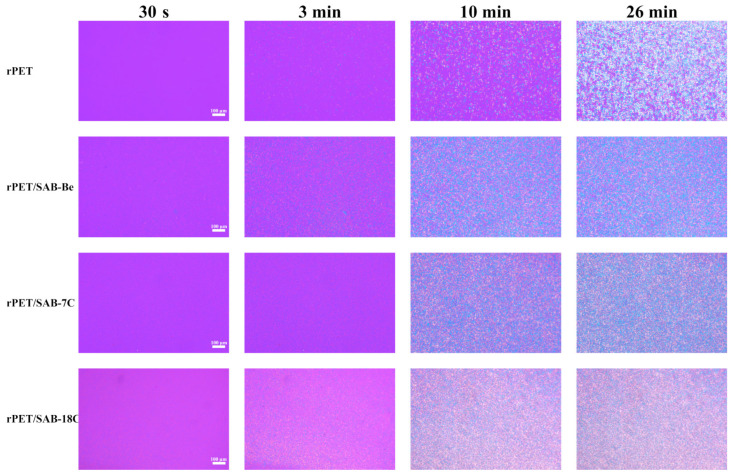
POM micrographs of the crystal morphology for rPET/nucleating agent blends during isothermal crystallization at 225 °C.

Figure 7 shows the crystal morphology of the samples during isothermal crystallization at 225 °C. It is clear that the addition of nucleating agents promotes spherulite growth in rPET, consistent with its isothermal crystallization behavior. Observation of the Polarized Optical Microscopy (POM) image of the rPET/nucleating agent blends at 26 min reveals sparse, large spherulites in rPET, whereas the blend exhibits a uniformly distributed fine crystalline structure. This indicates that nucleating agents increase nucleation density and refine spherulite size, effectively improving rPET crystal uniformity and thereby enhancing mechanical properties.

### 2.6. Lubrication Mechanism of the rPET/Nucleating Agent Blends

**Figure 8:**
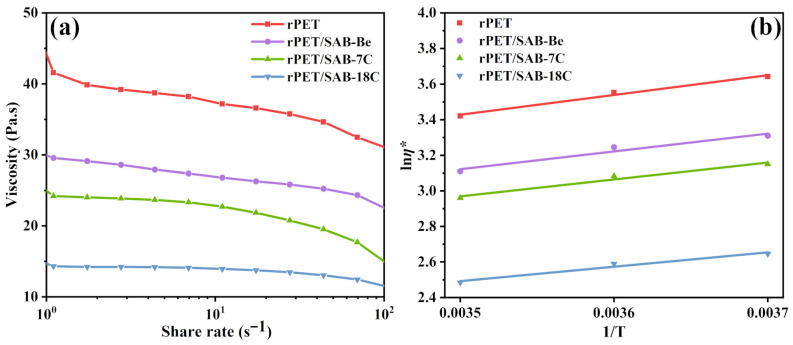
(**a**) Relationship between viscosity and shear rate of the rPET/nucleating agent blends at 270 °C; (**b**) relationship between viscosity and temperature of the rPET/nucleating agent blends.

The relationship between viscosity and shear rate of rPET/nucleating agent blends at 270 °C is shown in Figure 8a. It can be observed that under identical shear rates; the viscosity of the samples followed the order rPET > rPET/SAB-Be > rPET/SAB-7C > rPET/SAB-18C. The phenomenon demonstrates that nucleating agents can facilitate the rearrangement of the rPET molecular chains in the shear direction. Furthermore, nucleating agents with longer alkyl chains can provide lubrication at the nucleating agent–matrix interface, thereby further enhancing the mobility of rPET molecular chains.

The rheological properties of the rPET/nucleating agent blends required for further investigation using the Arrhenius equation:

(8) $$\begin{array}{c} \eta^{*}\;=\;Aexp\frac{∆E}{\mathrm{RT}} \end{array}$$

where η* was viscosity, Δ*E* was the viscous flow activation energy, *A* was the viscosity constant, *R* was the gas constant (8.314 J/mol∙K), and *T* was the shearing temperature.

According to Equation (8), lnη* was plotted against 1/*T*, with a slope of Δ*E*/*R* and an intercept of ln*A*. As shown in Figure 8b and Table 4, lnη* exhibited a clear linear relationship with 1/*T*, indicating that the melt flow of the rPET/nucleating agent blends fully conformed to the Arrhenius equation [45]. The order of ln*A* and Δ*E* for the samples was as follows: rPET > rPET/SAB-Be > rPET/SAB-7C > rPET/SAB-18C. The results indicate that SAB-18C provides an internal lubricating effect, effectively reducing the intermolecular forces between rPET chains and enhancing their flexibility. Without compromising the overall properties of rPET, it partially relieves local chain entanglements, thereby significantly improving the crystallization behavior of rPET.

### 2.7. Mechanical Properties of the rPET/Nucleating Agent Blends

**Figure 9:**
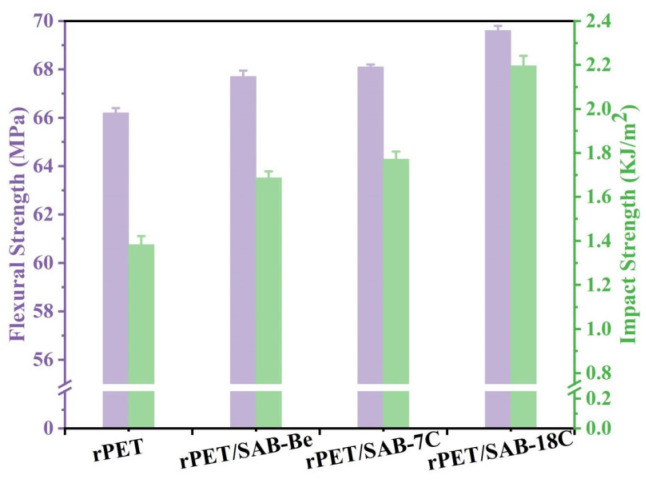
Mechanical properties of the rPET/nucleating agent blends.

As shown in Figure 9, compared to rPET, the flexural strength of rPET/SAB-Be, rPET/SAB-7C, and rPET/SAB-18C increased by 1.5 MPa, 1.9 MPa, and 3.4 MPa, respectively. The results indicate that as the compatibility between nucleating agents and rPET improves, the modification effect becomes more increasingly pronounced. *X*c value data corroborate this trend—increased Xc values enhance molecular chain alignment, thereby expanding crystalline regions and improving rPET flexural strength [46]. Furthermore, the improved compatibility between nucleating agents and rPET enhances the effect of the nucleating agents, making the nucleating agents not easy to introduce defects in rPET. This is beneficial for the formation of a dense crystal layer in rPET, thereby improving the flexural strength.

Meanwhile, the impact strength increased by 23.0%, 28.1%, and 58.9%, respectively. In POM testing, it was clearly observed that the addition of nucleating agents resulted in significantly smaller spherulite sizes in rPET. This refined crystalline structure enhances material toughness by strengthening interfacial bonding between spherulites, thereby inhibiting crack propagation and improving the impact strength of rPET. Furthermore, SAB-18C achieved a certain effect of plasticizing in rPET, further enhancing the impact strength.

### 2.8. Compatibility of the Nucleating Agents in rPET

**Figure 10:**
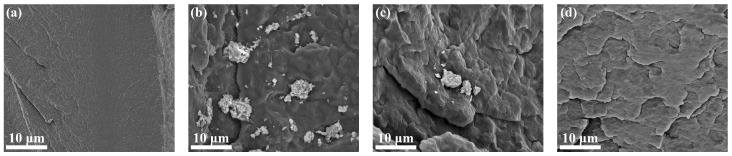
Fracture surface morphology of the rPET/nucleating agent blends. (**a**) rPET. (**b**) rPET/SAB-Be. (**c**) rPET/SAB-7C. (**d**) rPET/SAB-18C.

As shown in Figure 10, the SEM observations of the rPET/nucleating agent blends revealed conspicuous white particulate matter aggregating on the fracture surfaces of the rPET/SAB-Be and rPET/SAB-7C, with the phenomenon more pronounced on the rPET/SAB-Be surface. The resulted in reduced adhesion of SAB-Be to the rPET surface. The rPET/SAB-Be blend was more prone to separation and defect formation when subjected to external forces, which affected the mechanical properties. The SAB-18C was uniformly dispersed in the rPET matrix without obvious particle protrusion, indicating good compatibility between SAB-18C and rPET. This was due to the long alkyl chain in SAB-18C, which improved the compatibility with rPET and contributed to the reason for the good mechanical properties of rPET/SAB-18C.

## 3. Materials and Methods

### 3.1. Materials

The rPET (PCR80AP) was obtained from Ningbo Jianfeng New Material Co., Ltd. (Ningbo, China). 4-Aminobenzoic acid (PABA), benzoyl chloride, heptanoyl chloride, and stearoyl chloride were purchased from Shanghai Macklin Biochemical Co., Ltd. (Shanghai, China). Sodium hydroxide (NaOH) was supplied by Shanghai Aladdin Biochemical Technology Co., Ltd. (Shanghai, China). Acetone and ethanol were provided by Tianjin Tianli Chemical Reagent Co., Ltd. (Tianjin, China).

### 3.2. Preparation of the Nucleating Agents

**Figure 11:**
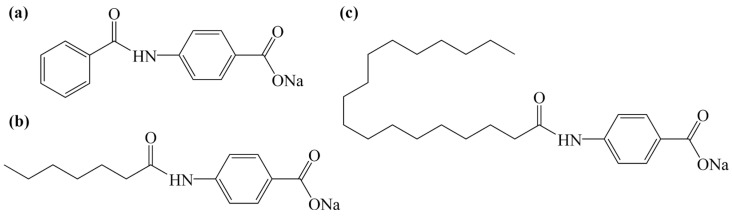
The molecular structures of nucleating agents (**a**) SAB-Be, (**b**) SAB-7C and (**c**) SAB-18C.

The initial step was to dissolve PABA (0.05 mol) in acetone (70 mL). Benzoyl chloride (0.025 mol) was then added dropwise and the reaction mixture was stirred for 7 h after the addition was complete. The solid product was obtained by suction filtration and washed with acetone and deionized water until the pH was close to 7. Subsequently, the product from the first step was stirred with 50% NaOH solution (0.05 mol) in deionized water (100 mL) for 8 h. The resulting crude product was also obtained by suction filtration and washed with deionized water until the pH was close to 7. Finally, the crude product was dried at 105 °C for 4 h to obtain sodium 4-[(benzyl)amino] benzoate (SAB-Be, Figure 11).

For the synthesis of sodium 4-[(heptanoyl)amino] benzoate (SAB-7C), the above procedure was followed using heptanoyl chloride instead of benzoyl chloride. In the neutralization step, acetone (100 mL) was used as the dispersion medium, and the product obtained was dried at 60 °C for 4 h. Sodium 4-[(stearoyl)amino] benzoate (SAB-18C) was prepared by replacing heptanoyl chloride with stearoyl chloride while keeping all other remaining experimental conditions unchanged. The molecular structures of the synthesized nucleating agents are shown in Figure 11.

### 3.3. Characterization of the Nucleating Agents

The FTIR spectra were recorded using a Nicolet iS10 spectrometer (Thermo Scientific, Waltham, MA, USA) with 64 scans per sample to confirm the chemical structures of the nucleating agents.

The TGA was conducted on a TGA-1 analyzer (Mettler Toledo, Zurich, Switzerland). Measurements were performed from 40 °C to 600 °C at a heating rate of 10 °C/min under a nitrogen atmosphere to evaluate the thermal stability of the nucleating agents.

### 3.4. Preparation of the rPET/Nucleating Agent Blends

The compositions of the rPET/nucleating agent blends are summarized in Table 5. The components were first remixed using a high-speed mixer (BL-500A, Songqing Hardware Factory, Yongkang, China) at 25,000 rpm for 2 min to ensure uniform distribution. Melt blending was carried out using a twin-screw extruder (WLG10A, Shanghai Xinshuo Precision Machinery Co., Ltd., Shanghai, China) at a processing temperature of 270 °C. The extrudates were pelletized and subsequently injection-molded into standard test specimens using an injection molding machine (WZS10D, Shanghai Xinshuo Precision Machinery Co., Ltd., Shanghai, China) at 270 °C with an injection pressure of 0.5 MPa.

### 3.5. Crystal Behavior of the rPET/Nucleating Agent Blends

Differential scanning calorimetry (DSC) measurements were conducted using a Q1000 DSC (TA Instruments, Milford, CT, USA) under a nitrogen flow of 50 mL/min.

For non-isothermal crystallization tests, samples were heated from 40 °C to 280 °C at 10 °C/min and held for 3 min to erase thermal history, then cooled to 40 °C at 10 °C/min. A second heating and cooling cycle was performed under the same conditions, with the final cooling rate increased to 20 °C/min.

Isothermal crystallization experiments were conducted by heating the samples to 280 °C and holding for 3 min, rapidly cooling to the selected crystallization temperatures at 40 °C/min, and maintaining isothermal conditions for 60 min. The samples were then reheated to 280 °C at 10 °C/min and cooled to 40 °C at 20 °C/min.

### 3.6. Polarized Optical Microscopy of the rPET/Nucleating Agent Blends

The crystal morphology of the rPET/nucleating agent blends during isothermal crys-tallization was observed using a polarizing microscope (BX51-P, Olympus Corporation, Tokyo, Japan) equipped with a thermostatic stage (THMS 600, Linkem Scientific Instru-ments Ltd., Oxfordshire, UK) under a cross polarizer. The sample was first heated to 280 °C and maintained for 3 min, and then cooled down to 225 °C with the cooling rate of 40 °C/min and held for 40 min. The crystal morphology of the sample could be observed at a constant temperature.

### 3.7. Rheological Properties of the rPET/Nucleating Agent Blends

The rheological behavior of the rPET/nucleating agent blends was evaluated using a rotational rheometer (DHR-2, TA Instruments, Milford, MA, USA) in oscillatory mode. A parallel plate geometry with a diameter of 25 mm and a gap of approximately 2 mm was used. Measurements were conducted at 270 °C, 275 °C, and 280 °C with a fixed frequency of 0.1 Hz.

### 3.8. Mechanical Properties of the rPET/Nucleating Agent Blends

The flexural properties were measured using a universal testing machine (TY-8000A, Jiangsu Tianyuan Test Instrument Co., Ltd., Yangzhou, China) in accordance with GB/T 9341–2008 at a crosshead speed of 5 mm/min. The Izod impact strength was determined using an impact tester (TY-4021A, Jiangsu Tianyuan Test Instrument Co., Ltd., Yangzhou, China) following GB/T 1043.1–2008. Tests were conducted with a pendulum energy of 5.5 J at a maximum release angle of 150°. Reported values represent the average of five specimens.

### 3.9. Compatibility of the Nucleating Agent Blends in the rPET Matrix

The dispersion and compatibility of nucleating agents within the rPET matrix were examined by SEM (Quanta 250 FEG, FEI Company, Hillsboro, OR, USA). Samples were cryogenically fractured in liquid nitrogen, sputter-coated with gold, and observed to analyze the fracture surface morphology.

## 4. Conclusions

In this study, the nucleating agents SAB-Be, SAB-7C, and SAB-18C, each with different alkyl chain lengths, were synthesized. The results of the FTIR analysis indicated that the nucleating agents were successfully synthesized. The TGA demonstrated that the nucleating agents did not decompose at the processing temperature of rPET, indicating that the SAB-Be, SAB-7C, and SAB-18C can be used as dispersed nucleating agents in rPET. The rPET/nucleating agent blends were prepared by extrusion. The non-isothermal crystallization behavior of the rPET/nucleating agent blends showed that the addition of nucleating agents provided heterogeneous nucleation sites for rPET, thereby improving the crystallization properties. In addition, the *T*_g_ of rPET, rPET/SAB-Be, rPET/SAB-7C, and rPET/SAB-18C were 80.3 ± 0.3 °C, 80.4 ± 0.9 °C, 77.0 ± 1.2 °C, and 69.7 ± 0.9 °C, respectively, indicating that increase in the alkyl chain length of nucleating agents can promote the mobility of rPET molecular chains. The isothermal crystallization behavior of rPET/nucleating agent blends indicates that increasing the alkyl chain length in the nucleating agent effectively reduces the nucleation energy barrier of rPET, thereby accelerating the crystallization rate. POM results corroborate this finding, with nucleating agents possessing longer alkyl chains exhibiting a more pronounced effect in refined spherulite size. Mechanical properties testing and SEM analysis indicate that the alkyl chain segments within the nucleating agent provide a degree of lubrication, enhancing compatibility between the nucleating agent and rPET, thereby delivering superior mechanical properties. In addition, long-term thermal stability, recyclability over multiple processing cycles, and aging behavior of the modified rPET were not addressed in this work. In the future, research will be conducted on these aspects to fully evaluate the industrial applicability of the nucleating agents.

## Figures and Tables

**Table 1:** Non-isothermal crystallization parameter of the rPET/nucleating agent blends.

Sample	*T*_g_ (°C)	*T*_cp_ (°C)	*T*_m1_ (°C)	*T*_m2_ (°C)	Δ*H*_m_ (J/g)	*X*_c_ (%)
rPET	80.3 ± 0.3	192.9 ± 2.1	238.8 ± 1.4	249.9 ± 0.6	32.1 ± 3.2	22.9 ± 2.2
rPET/SAB-Be	80.4 ± 0.9	209.8 ± 1.1	244.9 ± 0.3	250.3 ± 1.0	39.8 ± 1.7	28.4 ± 1.2
rPET/SAB-7C	77.0 ± 1.2	210.3 ± 0.6	245.4 ± 0.9	251.1 ± 0.4	40.2 ± 0.5	28.7 ± 0.4
rPET/SAB-18C	69.7 ± 0.9	213.8 ± 1.2	246.9 ± 1.0	-	42.0 ± 1.1	30.0 ± 0.8

**Table 2:** Isothermal crystallization kinetics parameter of the rPET/nucleating agent blends.

Sample	*T*_c_ (°C)	*t*^a^_1/2_ (min)	*n*	*K* (min^−n^)	*T*^b^_1/2_ (min)	*T*_m_ (°C)
rPET	221	7.0	2.49	0.0052	7.1	249.4
	223	14.0	2.65	0.0004	16.8	249.7
	225	17.6	2.79	0.0002	18.4	249.8
rPET/SAB-Be	225	3.6	3.01	0.0144	3.6	251.0
	227	4.9	3.25	0.0047	4.7	251.6
	229	8.6	3.22	0.0006	9.0	251.7
rPET/SAB-7C	225	3.1	3.00	0.0242	3.1	249.7
	227	3.7	3.01	0.0148	3.7	250.8
	229	7.4	3.30	0.0009	7.5	251.3
rPET/SAB-18C	225	2.9	3.19	0.0257	2.8	250.0
	227	3.6	3.33	0.0100	3.5	251.2
	229	6.0	3.41	0.0015	6.1	252.4

**Table 3:** Isothermal crystallization thermodynamic parameter of the rPET/nucleating agent blends.

Sample	*T*^0^_m_ (°C)	Δ*E* (KJ/mol)	*K*_g_ (K^2^)	*σ*_e_ (erg/cm^2^)
rPET	252.1	478.7	1.13 × 10^5^	21.0
rPET/SAB-Be	256.5	468.6	0.79 × 10^5^	14.7
rPET/SAB-7C	266.1	457.8	0.81 × 10^5^	15.0
rPET/SAB-18C	289.1	398.9	0.69 × 10^5^	12.8

**Table 4:** Arrhenius equation parameters of the rPET/nucleating agent blends.

Sample	*lnA* (Pa·s)	Δ*E* (J/mol)	*R* ^2^
rPET	0.46	133.5	0.988
rPET/SAB-Be	0.38	120.3	0.959
rPET/SAB-7C	0.36	114.3	0.972
rPET/SAB-18C	0.34	97.4	0.969

**Table 5:** The formula of the rPET/nucleating agent blends.

Sample	rPET (g)	SAB-Be (g)	SAB-7C (g)	SAB-18C (g)
rPET	200	-	-	-
rPET/SAB-Be	200	1	-	-
rPET/SAB-7C	200	-	1	-
rPET/SAB-18C	200	-	-	1

## Data Availability

Data are contained within the article.
